# King Edward's Hospital Fund for London

**Published:** 1918-05-18

**Authors:** 


### KING EDWARD'S HOSPITAL FUND FOR LONDON.

### Its Majority. Some Wonderful Results.

This Fund has attained its majority and held its twenty-first annual meeting, at St. James's Palace, on the 14th instant. It was fitting and grateful that the Speaker, the Right Hon. James W. Lowther, an ideal chairman, should be the presiding governor. Twenty-one years' experience of the administration and results achieved through King Edward's Fund have won, as they undoubtedly deserve, the gratitude and appreciative recognition of all knowledgeable people who take a heartfelt interest in the voluntary hospitals and everything that tends to promote their development and success.

The grants for 1917 amounted to £190,000, being £20,000 more than in 1916, and £32,500 in excess of the grants made in the two previous years of the highest distribution before the war. £181,000 was given to London hospitals and £9,000 to consumption sanatoria and convalescent homes taking London patients—a marked increase over 1916. Viscount Astor's munificence enabled the grants for maintenance to exceed those in 1915 by £34,650, and those in 1916 by £8,750. The total sum distributed among hospitals, convalescent homes, and consumption sanatoria since the foundation of the Fund approaches two millions and a half, whilst the annual distribution for the whole twenty-one years has been £117,067. It is interesting in this connection to note that £80,000 has to be raised each year from subscriptions, donations, and legacies to maintain the annual distribution at £190,000. It has been a rule of the Fund for two visitors, of whom one was a medical man and the other a layman, to inspect all the sanatoria receiving grants every year. Since the war, in view of the restrictions on travelling by motor and by rail, these inspections have had to be suspended.

We have not space to go fully into the whole of the figures, but the following have a special interest in war-time. The average number of beds at the London hospitals in daily occupation by naval and military patients in 1916 was 2,855; the total ordinary expenditure of the London hospitals, which in 1913 was £1,142,938, had risen in 1916 to £1,513,834, an increase of £370,896. In so far as this increase was due to the treatment of naval or military patients, it was partially covered by contributions from the State amounting to £218,087, the balance, amounting to £152,809, representing the additional ordinary expenditure which fell on the funds of the voluntary hospitals of London during 1916. This represents an increase of 13.36 per cent., compared with that of 1913. Supporters of hospitals, and benevolent donors, will realise from these figures that several hospitals, being most essential institutions, have suffered a material reduction in their available funds.

In great Funds like this the amount spent on administration represents an important factor, and is an index to efficiency. During 1917 administration cost £3,460, or £1 19s. 0½d. per cent. of the total amount received, as compared with £3,161, or 19s. 4½d., in the previous year. The corresponding percentage for the whole period since the inauguration of the Fund has been £1 4s. 6d., or less than 3d. in every pound received, the average yearly outlay on administration being £2,866. It is instructive and interesting to note that the increased expenditure here shown is due mainly to additions to the remuneration of the staff and the greater cost of stationery, printing, etc., caused by the war. The Council has suffered the loss, by death, of a generous subscriber in the person of the Duke of Norfolk, one of the kindest, most lovable, and generous of men. The services as visitors of Sir George Alexander and Mr. Arthur James have also been lost through death. The war has made itself felt throughout the land. Out of the King's Fund office staff of six members three have been on active service, of whom one, Mr. Frank G. Simmons, has died of wounds in Palestine, and Mr. Victor H. Rushton has been wounded and is a prisoner in Germany. Hospital funds and hospitals, whose main purpose is to minister to the recovery of the sick and wounded, have had their victims, to the great loss of these institutions and to all connected with them.

The audited accounts well repay careful study. A new and most acceptable feature is the summary of the totals of receipts, expenses, and awards, with the average in each case, during the twenty-one years of this Fund's existence. The original estimates on which the first appeal was based have on the whole been more than justified by the results achieved. The purpose was to raise from £100,000 to £150,000 a year from those who had not heretofore regularly contributed to hospitals. The average yield of the Fund during each of the twenty-one years of its existence has been £234,186. The exceptions prove how conscientiously the conductors of the Fund have fulfilled the late King Edward's undertaking that it should not trench upon the ground occupied by the Hospital Sunday and Saturday Funds. This fact should stimulate the small contributors of 10s. and under to make it their business to give something each year to the League of Mercy, and the middle and upper classes throughout the Metropolis to realise that King Edward's Hospital Fund **needs many small subscriptions of one guinea upwards** to make good the losses arising from death and other causes. The great work the King's Fund has done for hospitals is so universally recognised that there must be many people, we are confident, who would gladly give **an annual subscription of from one to three guineas to the King's Fund if they knew how welcome these smaller subscriptions would be.**

